# Prioritizing the bottom line over people in a crisis: How leader behavior affects employee psychological distress under economic threat

**DOI:** 10.1371/journal.pone.0323415

**Published:** 2025-07-31

**Authors:** Sofija Pajic, Claudia Buengeler, Deanne N. Den Hartog, Diana Hanke-Boer

**Affiliations:** 1 Institute for Management Research, Radboud University Nijmegen, Nijmegen, Netherlands; 2 Amsterdam Business School, University of Amsterdam, Amsterdam, Netherlands; 3 Institute of Business, Kiel University, Kiel, Germany; 4 Institute of Psychology, University of Koblenz-Landau, Koblenz, Germany; Southeast University, CHINA

## Abstract

**Aim:**

This study examines the impact of direct supervisors’ leadership on employee psychological distress during crises. Specifically, it explores the relationship between people-oriented and bottom-line-mentality informed leader behaviors and employee psychological distress. The study further hypothesizes that employees’ sense of control functions as a psychological mechanism in this relationship and that this indirect effect is moderated by personal and national economic threat, both of which are particularly salient during crises.

**Method:**

A quantitative survey was conducted in three waves among 854 employees across Europe (Germany, Italy, the Netherlands and Spain) during the COVID-19 pandemic, a highly salient global crisis.

**Results:**

Findings indicate that employees with people-oriented leaders report lower psychological distress, mediated by an enhanced sense of control. In contrast, bottom-line-mentality informed leadership is associated with a diminished sense of control, and further with greater psychological distress. These effects are exacerbated under higher personal and national economic threat.

**Conclusion:**

This study advances understanding of how constructive and destructive leadership behaviors influence employees’ psychological well-being during crises, particularly under economic strain. The findings provide actionable insights for leaders, organizations, and policymakers on mitigating employee psychological distress through leadership strategies that foster a sense of control.

## Introduction

Crises are rare, unexpected, and highly disruptive events that can significantly impair organizational functioning. Due to their infrequency and unpredictability, organizational leaders often lack experience and preparedness in crisis management. However, rapid and decisive responses are typically required to mitigate their potentially substantial consequences [1]. In the past decades, a number of major crises have affected organizations and disrupted their activities (e.g., the Global Financial Crisis, the Eurozone Debt Crisis, the COVID‐19 pandemic, the Russo-Ukrainian War, and the Global Energy Crisis) [1].

Global crises create scarcities of essential resources, including time, healthcare services, medical supplies, personnel, and consumer goods, leading to profound economic and social consequences [2]. Scarcity theory posits that perceived shortages of these essential resources deplete cognitive capacity, limiting individuals’ ability to address other demands [3,4]. Specifically, experiencing scarcity reduces mental bandwidth—cognitive control and the capacity for higher-order decision-making [5] —resulting in the neglect of concerns perceived as less urgent [3,4]. For employees, global crises introduce a sudden surge in pressure, workload, and job insecurity, which further exacerbates scarcity [6]. For instance, during the COVID-19 pandemic, many companies were forced to fundamentally alter their operations, while employees faced disruptions such as reduced or irregular work hours, mandatory remote work, and the challenge of balancing professional and childcare responsibilities [7]. In such conditions, where employees’ minds are preoccupied with excessive demands and disruptions, their mental bandwidth can become taxed [8]. As a result, employees may struggle with feelings of overwhelm due to scarcity, uncertainty, and workplace changes, impairing their ability to prioritize long-term goals, such as maintaining health and psychological well-being [9]. Hence, it is unsurprising that crises adversely impact employees’ psychological well-being, as evident in elevated levels of depression, worry, and distress [10,11]. Moreover, as observed in previous crises, the psychosocial impact often persists beyond the immediate crisis period [12]. Given the tremendous impact of crises (e.g., COVID-19 pandemic) on people’s personal and work lives, understanding workplace factors that might contribute to or mitigate potential declines in psychological health is vital for managing their short- and long-term psychological consequences [13,14]. In such circumstances, a stabilizing organizational presence is essential, as its absence may further exacerbate issues related to employee health and well-being [15,16].

One of the most influential stabilizing forces within organizations is leadership, as direct supervisors play a crucial role in shaping employees’ psychological well-being and their ability to cope with crisis-induced stress [17]. A substantial body of organizational research highlights the critical role of direct leaders in shaping employees’ psychological well-being. Leaders can foster well-being through constructive behaviors such as providing support, inspiration, and motivation [18], or conversely, they can exacerbate psychological distress through destructive behaviors such as aggression, abuse, or exploitation [19]. Further building on scarcity theory, which also states that individual responses to scarcity can be shaped by contextual factors [3,4], we argue that the role of direct supervisors’ leadership as the stabilizing agent for employee psychological health is even more important during crisis as they have less capacity to maintain their well-being themselves. Specifically, in such periods, employees are likely to experience attentional tunneling, focusing predominantly on immediate threats and resource shortages (e.g., health risks associated with COVID-19), making their well-being more at risk [3,4]. Consequently, leaders who help alleviate perceptions of scarcity can restore some of the cognitive capacity that is otherwise depleted by these conditions [20]. Accordingly, evidence from social network research shows that leaders play a crucial role in crisis coordination by effectively filtering misinformation and ensuring the dissemination of accurate and relevant information during crisis events [21].

However, leaders themselves do not operate in isolation; rather, their behaviors are also shaped by context [22]. This suggests that crisis conditions can trigger leaders to behave in certain ways, with their actions having substantial implications for employees [1,23]. To better understand leadership response in crises, threat rigidity theory can complement scarcity theory in explaining how leaders may act in the circumstances of external threats [24,25]. According to threat rigidity theory, leaders under threatening conditions, often resort to simplifying decision making, limiting communication, and imposing stricter control [24]. These rigid responses, while intended to stabilize organization, may inadvertently exacerbate employee stress and reduce their well-being in crises. Hence, since crises frequently jeopardize an organization’s financial stability, some leaders may prioritize economic survival over other concerns [26]. In other words, they may show bottom-line-mentality informed behaviors (BLM-informed), characterized by tightened control, centralized decision-making, and an overwhelming focus on securing performance outcomes [27]. Such leaders may neglect competing priorities, including employee support and consideration, under the assumption that maximizing performance is the key to organizational survival- often at the expense of employee well-being. In that sense, BLM-informed leadership could be construed as a negative and destructive form of task- or goal- oriented leadership [28]. While both styles emphasize performance, BLM-informed leadership can become a rigid, excessive form of task-oriented leadership that undermines motivation and well-being. On the contrary, for other leaders, the aspects of their leadership role which include caring and taking responsibility for others [29] might be more salient in crisis; such leaders might thus respond to crises with a strong concern for their employees and a focus .on providing support, avoiding layoffs, and minimizing the impact on employees [6]. Consistent with research on the benefits of considerate leaders for employees’ psychological health [30,31], leader’s people oriented behavior in the form of care and support can particularly alleviate employees’ psychological distress in a crisis [32]. Accordingly, business ethics research argues that crisis management needs to encompass ethics of care considerations, because a strictly economic approach may produce greater resentment and reputational damage [33,34]. Thus, we hold that a leader’s sole focus on the bottom line may be particularly troublesome for employees’ [27] psychological health during a crisis given the scarcity-induced vulnerability.

Furthermore, we propose that employee’s sense of control (or lack thereof) serves as a critical mechanism linking leadership behavior to psychological health. When leaders exert excessive pressure on employees in already resource-scarce conditions, employees may feel incapable of managing their workload, leading to heightened stress and helplessness [35]. Additionally, leaders who adopt a rigid, exhibiting a singular focus on the bottom line, tightening control and limiting employee involvement to safeguard financial stability, may inadvertently exclude employees from decision-making, increase uncertainty, and further erode their sense of control over their work [25]. As a result, employees feel even more resource-deprived, experience greater cognitive overload, and suffer a diminished sense of personal agency. Finally, BLM-informed leader behavior promotes ‘everyone for themselves’ thinking, thereby harming collaborative efforts to tackle stressors, further reducing employees’ sense of control and increasing distress [36]. In contrast, leader’s people oriented behavior during a crisis counters these effects providing resource support and sense of control as it offers a compass for orienting in an uncertain world and cultivates a sense that ‘we are all in this together’ [37], which in turn should lessen employee distress [37,36]. Supportive leaders help employees manage stress, reducing mental bandwidth depletion. They offer clarity, reassurance, and flexibility, fostering greater psychological control over work situations. When supported by their leader, employees feel that they are in control, that they matter, and that they are empowered to make a difference [31,34].

**Fig 1:**
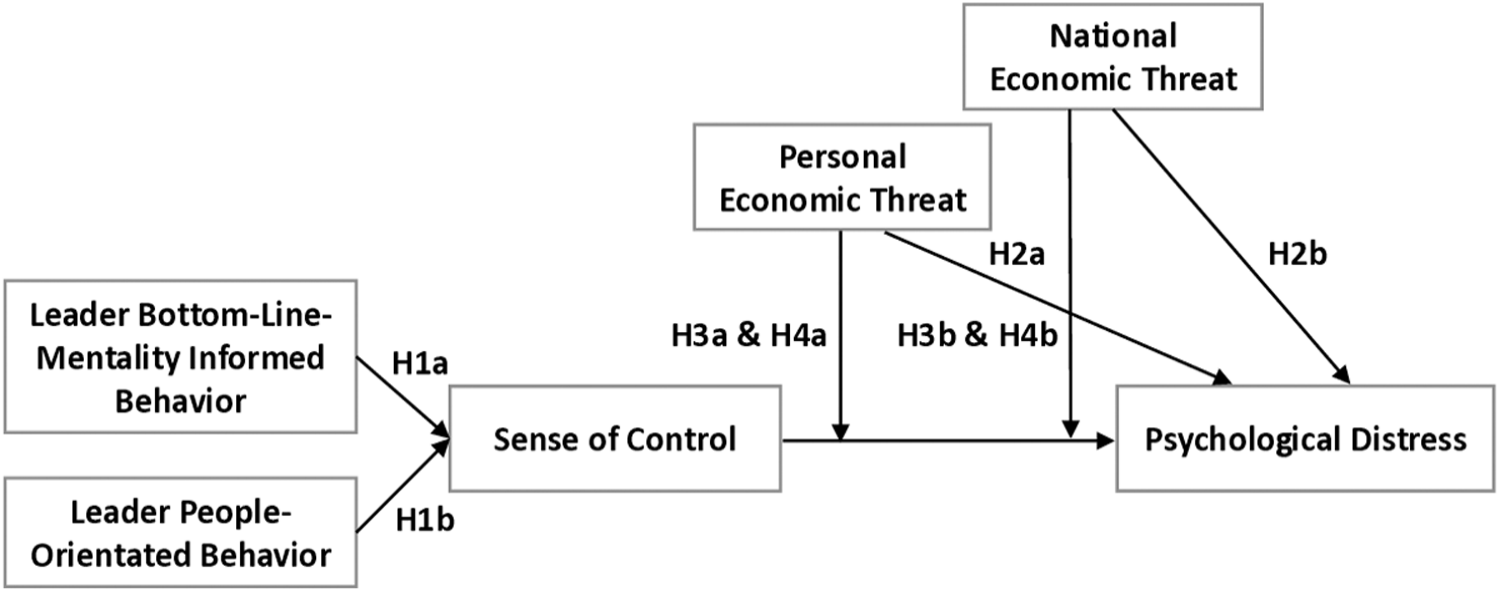
Conceptual model and the hypotheses of the current study.

Finally, we argue that employees’ psychological distress will be particularly elevated when employees also experience high economic threat during a crisis [38]. Because leadership is more impactful in times of higher uncertainty [39,40] and for employees with a higher need for leader guidance [41], employees experiencing economic threat during times of crisis might be particularly in need of support and care from their leaders to feel in control and avoid psychological distress [42], whereas they might be more negatively affected and feel less in control when their leaders single-mindedly pressure them to perform. The visual representation of our research model and hypotheses is depicted in Fig 1.

In sum, the current study aims to test the proposed opposing effects of BLM-informed versus people oriented behaviors on employee symptoms of psychological distress during crisis. We examine the mediating role of employee sense of control, and we test a second-stage moderation by which experiencing more pronounced economic threat would amplify the indirect effects of the two ways in which leaders can behave on psychological distress via sense of control. As a crisis context, the study was conducted at three measurement time points and in multiple countries during COVID-19. We focus specifically on the pandemic as a salient and global crisis, with high potential to affect employees’ well-being as well as the economic outlook of employees and organizations across the globe [6]. Our contribution is threefold. First, we add to work on leadership as a double-edged sword by studying the opposing psychological health effects of two prevalent ways in which leaders might behave during global crises, namely expressing concern for employees versus profits. While we know constructive forms of leading have health-promoting effects, less is known about the effects of negative forms of leading [43]. Even in the existing research on the impact of leaders on employees during the COVID-19 pandemic, the focus has been on constructive leader conduct, such are inclusive [16] and health-oriented leadership [31], while potentially destructive behaviors and their effects relative to constructive leadership have been understudied. Here, we test whether BLM-informed leader behavior during a crisis can harm employees’ psychological health, especially when they feel under threat. Second, we draw from scarcity theory to go beyond the thus-far scattered and undertheorized insights into the important role of leadership in vulnerable employees’ outcomes, and we introduce employees’ sense of control as a powerful mechanism in the relationship between leadership and distress in times of scarcity. Third, answering calls to address contextual factors in leadership [43–45], we study perceived personal and national economic threat as explicit boundary conditions that amplify the scarcity-induced vulnerability.

## Literature review

### Leader behavior and employee psychological distress during a crisis

We focus here on two forms of leader behavior that seem likely to occur during a crisis, as leaders themselves are also faced with a situation of scarcity where their attention narrows to maximizing chances of organizational survival. During a crisis, leaders may react with rigidity and pressure to perform [46] and focus solely on the bottom line, or they may show consideration and other actions that reflect care for the welfare of employees [22,32]. Actions that express an orientation towards people in the form of care and support during a crisis can alleviate employees’ psychological distress. In contrast, focusing exclusively on the bottom line may be particularly harmful to employee psychological health in such times. We argue that this is the case especially because, through the orientations they display, leaders affect employees’ sense of control in the work domain, which is particularly important in times of scarcity [47].

Sense of control, or individuals’ perceived ability to recognize, prevent, and manage potential harm [48], captures individuals’ beliefs about how much they can influence the course of their own outcomes [49]. A sense of control enables individuals to cope with adversity [50] and is linked to psychological health. Representing an important resource in the work domain [51], sense of control has been theorized as an essential psychological mechanism linking social and working conditions to individual mental health [52]. Here, we propose a crucial role of leader behavior during crisis in employees’ psychological health via increasing or decreasing their sense of control at work.

Social resources can be used to sustain sense of control and serve a stabilizing function in times when resources are generally scarce [53]. In times of crises and altered working conditions, employees’ own self-control resources are taxed, making them more receptive to information provided by others, such as their leaders [54], and more likely to agree with their leaders [55]. Accordingly, in crises, leaders might become an important source support or hindrance of employees’ sense of control at work. Namely, leaders whose behaviors reflect prioritizing the bottom line, and who thus react with pressure during a crisis, are likely to undermine employees’ sense of control. Although focusing on the bottom line might enhance productivity and support immediate organizational survival during times of scarcity [56], it can become problematic when employees are treated as if profit were the organization’s only objective [57]. While research on bottom line mentality has mainly focused on its effects on employee behaviors [58,59], this orientation has also been found to relate negatively to employees’ feelings and attitudes, such as diminished psychological safety and psychological health [27]. Leaders who display BLM-informed behavior pressure employees to perform, punish employees who are not up to par, and are willing to ignore ethical practices or employee needs to maximize profit [58]. These leaders base their decisions on immediate financial gain and the value of bottom line achievements for self-serving purposes [60], without considering the long-term implications for employees. When leaders show unidimensional thinking revolving around securing profit and productivity during crises [27], this reveals to employees that their valid concerns might be treated as irrelevant and illegitimate. As a result, employees may feel a lack of control and increased psychological distress [61].

In contrast, leaders who prioritize people and show consideration are likely to foster employees’ sense of control, as supportive interactions between employees and leaders instill control beliefs that employees further internalize through role modeling [62]. Consideration refers to a leader’s efforts to show concern for employees’ well-being, express support, and display warmth and approachability [63]. Leaders who are considerate, are supportive, and prioritize employee development and growth may be more sensitive to employees’ needs and thus more likely to seek to meet their basic needs for autonomy, competence, and relatedness, which are important for maintaining sense of control at work [64], particularly during times of crisis [65]. This can protect employees from feelings of hopelessness, worry, and loss of meaning [66]. In times of crisis, individuals tend to experience an enhanced need for security and belongingness, which supervisors oriented toward people are likely to meet [e.g., [67]. In addition, leaders who encourage employees, help them get motivated, and enhance their skills through training and development can help to strengthen employees’ employment sustainability in times of crisis, especially among more engaged employees [68]. Also, leaders can provide emotional and material support, which reduces strain [69], further strengthening employees’ sense of control and thereby reducing their psychological distress. Leader guidance and individualized support have been found to reduce employee strain in critical situations [70]. In sum, leaders whose actions support their employees’ sense of control protect their employees’ psychological health, whereas leaders whose actions undermine employees’ sense of control are likely to harm it. We hypothesize:

*Hypothesis 1: Bottom line mentality informed leader behavior relates positively (1a), and people oriented leader behavior relates negatively (1b), to employee psychological distress through employee sense of control.*

### The moderating role of economic threat

We have hypothesized that, in crises, direct supervisors’ behaviors that reflect orientation toward people or toward the bottom line relate to employees’ psychological distress by affecting their sense of control at work. Next, we complement the main proposition of scarcity theory with a threat-theoretical perspective [38,71], which we use to argue that the indirect relationship we have proposed is even stronger when scarcity-related vulnerability due to the crisis is accompanied by high (vs. low) economic threat. According to threat theorizing [38,71], a realistic threat refers to a concrete attack on physical well-being and economic resources [e.g., 60]. In global crises, the main sources of realistic threat are always coupled with economic stagnation, as crises are found to be among the strongest (if not the strongest) predictors of low economic productivity [38]. For example, for many individuals and groups, the COVID-19 pandemic yielded not only a strong realistic threat through its attack on physical health but also an economic threat to material resources, such as possessions (e.g., money) and income sources (e.g., employment) [72]. People may perceive this threat on a personal level (e.g., when they fear personal economic descent) and/or the national level (e.g., when they perceive their country as suffering an economic crisis) [73]. Here, we propose and empirically test how these two forms of economic threat—that is, personal and national economic threat—affect employee psychological distress and how they shape the indirect effect of the leader behavior on psychological distress through sense of control.

First, personal economic threat refers to individuals’ concerns about the security and stability of their personal finances [74]. Because personal finances are important for securing livelihood and maintaining standards of living, any potential destabilization of personal finances or fear of such destabilization impacts individuals negatively. Experiencing economic threat is in itself connected to negative health symptoms [75,76]. Moreover, some people may feel particularly fearful, uncertain, and preoccupied about their finances during global crises, because the conditions of the crisis may lower their ability to meet monthly living expenses. In addition, under higher perceived threat, individuals exercise less self-control [77], show less reappraisal of threatening events, and engage in more suppression of emotions [78]. They also tend to deploy more dysfunctional coping mechanisms [79], which might contribute to developing symptoms of psychological distress. Accordingly, research suggests that employees facing economic threat in the form of job insecurity [80] and financial insecurity [81] are at higher risk of adverse health outcomes. Second, individuals do not appraise economic threat exclusively in the context of their own financial status but are likely to also consider macro-economic influences, and their feelings and behavior are affected by the broader economic context in which they work [78]. A systematic review of the mental health outcomes of economic recessions found that periods of global economic hardship were associated with a higher prevalence of common mental disorders and suicidal behavior [82]. However, global crises and economic downturns tend to have less adverse effects on health in high-income countries and more adverse effects in countries with higher unemployment and lower employment protection [83]. In particular, the COVID-19 pandemic is an illustrative case of how a global crisis triggers varying economic consequences and remediating measures, including higher unemployment rates, decreases in working hours, layoffs, non-renewal of many temporary contracts, and hiring freezes. These effects were estimated to be ten times greater on average than the effects observed in the first months of the global financial crisis in 2008 [3]. For example, at the onset of the pandemic, more than one third of Europeans (38%) expected their financial situation to deteriorate, with 27% reporting having no savings to buffer income losses [78]. Similarly, among workers who still had a paid job in early April 2020, 35% in the US and 30% in the UK reported lower earnings compared to earlier months [79]. Government measures and institutional structures are essential to regulate the financial system in such situations [84]. Governmental policies that alleviate the causes of economic threat (e.g., by controlling and regulating minimal working hours or by providing financial support in case of layoff) may relieve some feelings of psychological distress in threatening economic conditions [20], whereas policies that create additional burdens may be potentially detrimental to employees’ psychological well-being. Therefore, in addition to personal economic threat, we argue that perceived national economic threat (i.e., the employee’s concerns about their country’s economic recession due to a crisis) also relates to their feelings of psychological distress. We hypothesize:

*Hypothesis 2: Economic threat, in the form of personal economic threat (2a) and national economic threat (2b), relates positively to psychological distress.*

Earlier, we argued that because crises substantially alter the ways in which work is performed, supervisors whose behavior help or hinder employees’ sense of control will particularly influence employees’ psychological distress. Importantly, as economic threat in crisis augments uncertainty, it also enhances the importance of sense of control [85] as well as the importance of leaders for followers [22,40]. More specifically, because economic threat challenges people’s self-concept [86], employees in a position of economic threat will increasingly tend to evaluate themselves and the circumstances using contextual cues, such as input from their leader, as these employees will be less certain about the appropriateness of their attitudes and behaviors [87]. The behavioral plasticity effect [88] supports this by showing that contextual cues are more salient for more vulnerable individuals. Employees who feel less economically threatened will be more equipped to deal with environmental cues from a personal security perspective, as they will not need to continuously adapt their attitudes and behaviors to the context [88] and will depend less on the input from the leader. Consequently, we expect that the sense of control derived from the leader’s behaviors may be even more impactful for the psychological health of employees experiencing higher personal and national economic threat, as they are in a more vulnerable position and feel more uncertainty than those whose economic situation is less strained. Employees with greater financial concerns often have lower socioeconomic status, less education, and lower job security. They frequently work in jobs with high physical and psychological demands [89] and have fewer resources to cope with these demands [90]. In addition, crises may shake up preexisting hierarchies and orders, in the sense that some industries and professions will be “winning” while others are losing.

Some empirical evidence suggests that, under the condition of economic vulnerability, the correlation between sense of control and mental health becomes more pronounced. Namely, Chou and Chi [85] found that sense of control may act as a coping mechanism, such that the economic strain is appraised in a less stress-inducing manner among (elderly) individuals with a higher sense of control. Similarly, economic threat had a weaker positive relationship with depression among (older) adults with higher (vs. lower) internal locus of control [91]. When experiencing personal economic threat, a reduced sense of control at work can be more devastating [92], as such a sense of control could have buffered against the felt threat of losing what little one has. Thus, leaders that reduce or enhance a sense of control will be more impactful for well-being of employees facing stronger economic threat. In contrast, employees with fewer financial concerns are less affected by leadership, as they are more equipped to process and deal with environmental cues by relying on their own control resources and might be less dependent on contextual cues in adapting to the current situation [88,93].

In sum, we expect that employees who experience greater personal economic threat are more vulnerable, which will enhance the potential for leaders to help prevent or promote symptoms of psychological distress among these employees by supporting their sense of control. Under conditions of threat, the sense of control facilitated by a considerate leader should figure importantly in reducing symptoms of distress, as it buffers against uncertainty and fear elicited by a worrisome financial situation. Likewise, higher personal economic threat should increase the detrimental consequences of BLM-informed leader behavior for psychological health by negatively affecting the employees’ sense of control given their heightened sensitivity to leaders. We hypothesize second-stage moderation:

*Hypothesis 3: Personal (3a) and national (3b) economic threat strengthen the indirect relationship of bottom line mentality informed leader behavior with employee psychological distress through sense of control.*

*Hypothesis 4: Personal (4a) and national (4b) economic threat strengthen the indirect relationship of people oriented leader behavior with employee psychological distress through sense of control.*

## Materials and methods

### Participants and procedure

The current study adopts a positivist research paradigm [94] to empirically test the relationship between leader behaviors and employee psychological distress in times of crisis. Building upon the propositions of scarcity theory, the study has taken a deductive approach in formulating theory-driven hypotheses about the relationships between the focal variables. The study relies on a quantitative, survey-based research strategy as a general approach of collecting the data.

Prior filling in the questionnaire, participants provided informed, written consent by indicating that they have read and understood the information about the purpose of the study, that they are aware that the data obtained will only be used anonymously, and that they give their consent for the obtained data to be used for research purposes. The data was analyzed fully anonymously. No minors or animals took part in the study. Written approval has been received from the Ethics Committee of the University coordinating the research. The ethics committee evaluated the research in terms of potential impact of the research on the human participants, the level and types of information and explanation provided to the participants at various stages of the research process, the team’s expertise in conducting the proposed analyses and particularly in terms of restricted access to the data to guarantee optimal levels of anonymity to the participants. The ethical approval is provided in the attachment to this submission.

Specifically, we collected panel data from paid participants residing in Germany, the Netherlands, Italy, and Spain. Participants had to be at least 18 years old, to have been employed at least part-time at the onset of the pandemic, and to have a direct supervisor. Because we collected data in several European countries that, at the time of the data collection, differed in both the current severity of the spread of the virus and their economic conditions prior to and during the pandemic, we group Italy and Spain into a higher-severity cluster and Germany and the Netherlands into a lower-severity cluster.

At Time 1, in early April 2020, 590 participants from Germany, 736 from the Netherlands, 684 from Italy and 645 from Spain filled out the survey. After excluding participants who did not meet the eligibility criteria or who failed the quality check questions, the final Time 1 sample included a total of 1335 participants: 331 from Germany, 336 from the Netherlands, 335 from Italy, and 333 from Spain. These individuals were invited to participate at Time 2, four weeks after Time 1. The response rate at Time 2 was 77.68%. The final Time 2 sample included 1037 participants, 277 participants from Germany, 251 from the Netherlands, 260 from Italy and 249 from Spain. Finally, the participants who filled in these two surveys were invited to participate in the data collection at Time 3, six months after Time 2. At Time 3, 854 participants responded (64.0%), 228 from Germany, 207 from the Netherlands, 218 from Italy, and 201 from Spain. We restricted the analyses to include only those respondents who took part in all three waves of data collection, because we were interested in the effects of leadership at Time 1 on psychological distress at Time 3, yielding a final sample size of N = 854. In total, 435 participants belonged to the lower-severity cluster, while 419 belonged to the higher-severity cluster.

To determine whether attrition produced any substantial difference in the variables of interest for this study, we examined whether the scores in the study variables measured at Time 1 differed between the participants who completed the first survey and those who took part in all three waves of data collection. Univariate analyses of variance were not significant for leader orientation toward people *F* (1,1333) = 1.833, *p* = 0.176 or for leader orientation toward the bottom line *F* (1,1333) = 1.494, *p* = 0.222. Thus, attrition did not appear to create bias regarding the primary variables of interest.

## Measures

Response options for most measures ranged from 1 (*fully disagree*) to 7 (*fully agree*). Measures for which no validated translation was available were translated into Dutch, German, Spanish, and Italian using a back-translation procedure. At Time 1, participants were instructed to answer items in their country for the situation since the onset of the spread of the COVID-19 virus. At Time 2 and Time 3, participants completed items focusing on the situation in their country since Time 1 and Time 2, respectively. We used shortened versions of the leadership scales, and we provide validity evidence from a separate sample for these short forms in the Supplementary Material.

### Leader behavior

Employees rated their leaders’ behavior at Time 1. Employees rated BLM-informed leader behavior using three items from a bottom line mentality scale [27]. A sample item is: “Treats the bottom line as more important than anything else.” Cronbach’s alpha was 0.82. We assessed people oriented leader behavior using three items from the revised Leader Behavior Description Questionnaire [LBDQ-XII; [95]]. A sample item is: “Looks out for the personal welfare of the team members.” Cronbach’s alpha was 0.92. To provide further assessment of the psychometric properties of the short forms of the measures used to assess leader behavior, we conducted a supplementary study and evaluated the measures’ reliability and factor structure, as well as the convergent validity between the short and the long forms. These results are presented in S1 Data Attachment.

### Sense of control

At Time 2, participants were instructed to answer three items adapted from Lachman and Weaver [52]. Participants were instructed to focus on how they feel about their work in the current circumstances since Time 1: “Under these circumstances, what happens in my life is beyond my control.” The original items were phrased negatively, measuring lack of control, so we recoded them prior to further analyses. Cronbach’s alpha was 0.82.

### Perceived personal and national economic threat

At Time 2, participants rated whether they personally experience personal financial concerns due to the pandemic by responding to a three-item scale [96] indicating, for example, how often they worry about being able to meet normal monthly living expenses since the onset of the spread of the COVID-19 virus in their country. Cronbach’s alpha was 0.84. Participants rated national economic threat using one item: “How threatening do you perceive the current situation related to the spread of COVID-19 in your country to be to national economic development?” Responses ranged from 1 (*not at all*) to 6 (*extremely*). We asked about the experience of economic threat at Time 2 to allow the participants some time to experience and evaluate the financial impact of the pandemic.

### Symptoms of psychological distress

At Time 3, symptoms of psychological distress were measured using eight items from the 9-item Patient Health Questionnaire [PHQ-9; [97]]. We excluded one item (“Thoughts that you would be better off dead or hurting yourself”) to avoid triggering suicidal thoughts during the crisis, especially given the higher suicide risk during the pandemic [e.g., [98]]. For the remaining items, participants indicated how often they experienced psychological problems over the past month, such as: “Little interest or pleasure in doing things.” Responses ranged from 1 (*not at all*) to 4 (*nearly every day*). Cronbach’s alpha was 0.92.

### Control variables

We controlled for factors that might affect how individuals felt during the pandemic and how they interacted with their leaders, including the country cluster, participants’ gender, age, education, childcare responsibility at home, ability to work from home, risk of severe COVID-19 symptoms due to their health status, contract type, working hours, being in a leadership position, tenure within the job and with the leader, gender of the leader, and the frequency of contact between employee and their leader after the start of the COVID-19 pandemic.

### Analytical strategy

We performed confirmatory factor analyses (CFA) and path analyses using MPlus (Version 7). A maximum likelihood estimator with robust standard errors (MLR) was used to test the measurement and hypothesized path models. Following standard CFA practices [99], we used several fit indices to assess our model fit. The robust comparative fit index (CFI) should be higher than.90, the robust root mean square error of approximation (RMSEA) and the standardized root mean square residual (SRMR) should be below.08 to indicate an acceptable fit [100]. Additionally, we computed 95% bias-corrected bootstrap confidence intervals for the indirect effect based on 5000 bootstrapped samples. We estimated the indirect and interaction effects within the same model (e.g., [101]) and tested the model with and without controls [102]. We plotted significant interactions with Johnson-Neyman plots using the MPlus to illustrate how the relationships vary across the values of continuous moderator variables (for how to do this see tutorial [103]).

## Results

### Descriptive statistics and correlations

Table 1 presents descriptive statistics, correlations, and reliability coefficients. Overall, correlations between BLM-informed leader behavior and psychological distress were significantly positive (r = .29, p < .001), whereas correlations between people oriented leader behavior and psychological distress were significantly negative (r = −.28, p < .001).

**Table 1:** Correlations and reliability of study variables.

	M	SD	1	2	3	4	5	6	7	8	9
1.Country cluster	.49	.50	(-)								
2.Gender	.51	.50	.07	(-)							
3.Childcare	.37	.48	.19**	−.02	(-)						
4.Age	46.76	1.67	−.08*	−.07*	−.19**	(-)					
5.Risk group	.22	.41	−.01	.02	−.02	.20**	(-)				
6.Education	2.33	1.02	.18**	.02	.10**	−.13**	−.01	(-)			
7.Contract	.28	.45	−.37**	−.01	−.10**	.08*	.03	−.06	(-)		
8.Position	.40	.49	.14**	−.06	.15**	−.03	.08*	.22**	−.09*	(-)	
9.Working from home	.43	.50	−.11**	.00	−.10**	.10**	−.01	−.09*	.10**	−.11**	(-)
10.Working hours	29.85	12.32	−.14**	−.01	−.05	−.08*	.03	.13**	−.02	.11**	−.15**
11.Job tenure	11.36	9.23	.11**	.01	−.08*	.49**	.07*	−.05	−.04	.01	.00
12.Frequency of contact	3.05	1.46	−.00	−.01	.03	−.08*	−.02	.01	−.09*	.20**	−.11**
13.Leader gender	.39	.50	−.14**	.16**	−.04	.02	.01	.01	.22**	−.08*	.05
14.Leader tenure	6.48	6.51	.11**	−.01	.01	.31**	−.00	−.04	−.13**	.11**	−.01
15.BLM-informed leader behavior	3.14	1.50	.24**	.02	.06	−.10**	−.00	.02	.00	.04	−.06
16.People oriented leader behavior	4.93	1.48	−.23**	−.05	−.02	.09*	−.00	.03	.12**	.10**	.03
17.Sense of control	4.29	1.42	−.10**	−.13**	−.05	.11**	−.00	−.05	−.03	.06	−.09*
18.Personal economic threat	2.49	.90	.21**	.09*	.07*	−.04	.03	−.13**	−.09**	−.15**	.07*
19.National economic threat	4.92	.96	.20**	.00	.11**	−.04	.06	.05	−.08*	.03	−.10**
20.Psychological distress	1.74	.70	.16**	.14**	.09**	−.16**	.09*	.07	−.10**	.05	.05
	**10**	**11**	**12**	**13**	**14**	**15**	**16**	**17**	**18**	**19**	**20**
1.Country cluster											
2.Gender											
3.Childcare											
4.Age											
5.Risk group											
6.Education											
7.Contract											
8.Position											
9.Working from home											
10.Working hours											
11.Job tenure	−.05	(-)									
12.Frequency of contact	.25**	−.05	(-)								
13.Leader gender	.02	.04	−.03	(-)							
14.Leader tenure	−.08*	.50**	.09*	−.05	(-)						
15.BLM-informed	.00	−.04	−.02	.01	−.04	(.822)					
16.People oriented leader behavior	.05	.06	.09**	.03	.11**	−.58**	(.924)				
17.Sense of control	.09**	.03	.08*	−.10**	.14**	−.24**	.25**	(.824)			
18.Personal economic threat	−.19**	−.04	−.11**	−.03	−.02	.18**	−.30**	−.20**	(.840)		
19.National economic threat	−.05	.00	−.02	−.06	−.02	.09**	−.07*	−.04	.05	(-)	
20.Psychological distress	−.07*	−.05	−.01	.00	−.07	.29**	−.28**	−.48**	.27**	.15**	(.923)

*Note. N* = 854 **p < .01 *p < .05. Reliability coefficients are presented in the diagonal. Country cluster was coded as 0 = Low severity cluster; 1 = High severity cluster. Gender was coded as 0 = Male; 1 = Female. Contract was coded as 0 = Permanent; 1 = Temporary. Position was coded as 0 = Non-managerial; 1 = Managerial. Belonging to a Risk group, Childcare, and Working from home were coded as 0 = No; 1 = Yes.

### Measurement model

We performed a set of CFAs to assess the discriminant validity of the constructs and compare the fit of the hypothesized five-factor measurement model to alternative models. The hypothesized measurement model adequately fit the data (SB-χ^2^ = 565.92, *df* = 160, *p* < .001; CFI = .95; RMSEA = .06; SRMR = .04) and fit better than all potential alternative models (Table 2).

**Table 2:** Confirmatory Factor Analysis Results for the Measurement Model.

Models	RMSEA	*CFI*	SRMR	SB-*χ2*	Df	SB-SCF	*Δ χ2/Δdf*	*ΔCFI*
1-factor	.17	.50	.15	4459.44	170	1.20	–	–
2-factor	.13	.73	.10	2495.99	169	1.17	586/1***	.25
3-factor	.11	.79	.12	1993.19	167	1.16	586.81/2***	.06
4-factor	.08	.89	.05	1076.81	164	1.16	205.91/3***	.11
5-factor (proposed model)	.06	.95	.04	565.922	160	1.15	713.44/6***	.08

Note: *N* = 845. 5-factor: each variable loaded on a corresponding first order factor. 4-factor: leadership variables loaded on a single construct, sense of control loaded on the second factor, personal economic threat loaded on the third factor, and symptoms of anxiety and depression loaded on the fourth factor. 3-factor: leadership variables loaded on a single construct, sense of control and personal economic threat loaded on the second factor, and symptoms of anxiety and depression loaded on the third factor. 2-factor: leadership variables loaded on a single factor, whereas personal economic threat, sense of control and symptoms of anxiety and depression loaded on the second factor. 1-factor: all variables loaded on a single factor. *p < .001.

To assess whether our measures were invariant across the two clusters, we conducted a multi-group CFA to test configural, metric, and scalar invariance. Following Chen [104], we evaluated changes in the model fit based on cutoff values for ΔCFI = .01 and ΔRMSEA = .015. The results indicated configural, metric, and scalar invariance of the measurement model (Table 3). As two scalar- and metric-invariant indicators are deemed sufficient for accepting measurement invariance [105], we proceeded with our hypothesis tests.

**Table 3:** Measurement Invariance Across Clusters.

Models	RMSEA	CFI	SRMR	SB-χ2/ SB-SCF	df	ΔRMSEA	ΔCFI	ΔCFI	ΔSRMR
Model 1 unconstrained (configural invariance)	.057	.946	.044	770.628***/1.13	320	–	–	–	–
Model 2 factor loadings (metric invariance)	.056	.947	.046	778.975***/1.13	335	.001	.001	.001	.002
Model 3 intercepts (scalar invariance)	.059	.939	.046	864.200***/1.13	350	.008	.003	.003	.000

Note: *N* = 854.

### Hypothesis testing

Overall, the models including the control variables fit the data better than those without, although the parameter estimates were nearly identical. As our controls offer information about the context, we kept them for model testing (Table 4). Specifically, in our path model, we regressed sense of control on BLM-informed leader behavior, people oriented leader behavior, and the control variables, and we regressed psychological distress on BLM-informed leader behavior, people oriented leader behavior, sense of control, personal economic threat, national economic threat, the product of sense of control and personal economic threat, the product of sense of control and national economic threat, and the control variables. The hypothesized model with controls fit the data well (*χ*^2^ = 13.31, *df* = 4, *p* = .010; CFI = .978; RMSEA = .05; SRMR = .02).

**Table 4:** Moderating role of economic threat on the relationships among leadership, sense of control, and psychological distress.

	Sense of control		Psychological distress
Independent variables			
BLM-informed leader behavior	−.14 (.04) **		.13 (.04) ***
People oriented leader behavior	.14 (.04) ***		−.06 (.04)
Mediating variable			
Sense of control	–		−.38 (.03) ***
Moderating variables			
Personal economic threat	–		.13 (.03) ***
National economic threat			.11 (.03) ***
Interaction terms			
Sense of control*Personal economic threat	–		−.10 (.03) **
Sense of control*National economic threat	–		−.08 (.03) **
Control variables			
Country cluster	−.04 (.04)		−.03 (.03)
Gender	−.10 (.03)**		.07 (.03) *
Childcare	−.03 (.03)		.02 (.03)
Age	.08 (.04)*		−.13 (.04)***
Risk group	−.12 (.03)		.09 (.03)**
Education	−.05(.03)		.03 (.03)
Contract	−.03 (.04)		−.09 (.03)**
Position	.03(.03)		.07 (.03)*
Working from home	−.09 (.02)**		.06 (03)*
Working hours	−.08 (.04)*		−.04 (.03) *
Job tenure	−.08 (.04)		.03 (.04)
Frequency of contact	.13(.03)		.03 (.03)
Leader gender	−.07 (.03)*		−.03 (.03)
Leader tenure	.13 (.04)***		.01 (.03)
R²	.34		.14
Indirect effects	Bootstrapped estimate	Boot 95% LLCI	Boot 95% ULCI
BLM-informed leader behavior	.054	.022	.087
People oriented leader behavior	−.055	−.087	−.023

Note: *N* = 845. ***p < .001 **p < .01 *p < .05. Values in parentheses are standard errors of model parameter estimates*.* Country was coded as 0 = Low severity cluster; 1 = High severity cluster. Gender was coded as 0 = Male; 1 = Female. Contract was coded as 0 = Permanent; 1 = Temporary. Contract was coded as 0 = Non-managerial; 1 = Managerial. Belonging to a Risk group, Childcare, and Working from home were coded as 0 = No; 1 = Yes.

Hypothesis 1a states that BLM-informed leader behavior relates positively to psychological distress through sense of control, and Hypothesis 1b states that people oriented leader behavior relates negatively to psychological distress through sense of control. For BLM-informed leader behavior, the indirect effect was positive (β = .05, p = .001; standardized bootstrapped estimate = .054; 95% CI [.022,.087]), while it was negative for people oriented leader behavior (β = −.06, p = .001; standardized bootstrapped estimate = −.055; 95% CI [−.087, −.023]). These results support Hypothesis 1a and 1b.

Hypothesis 2a states that there is a positive relationship between personal economic threat and psychological distress. In support of this hypothesis, the link between personal economic threat and psychological distress was indeed positive and significant (*β *= .13, **p* *< .001). Hypothesis 2b states that perceived national economic threat relates positively to psychological distress. Indeed, in support of this hypothesis, the relationship was positive and significant (*β *= .11, **p* *< .001).

**Fig 2:**
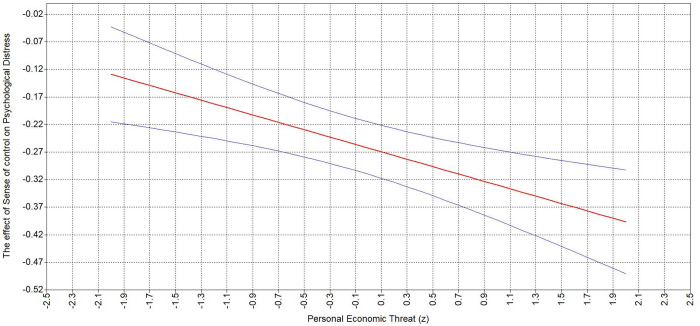
Johnson-Neyman plots depicting interaction between Sense of Control and Personal Economic Threat.

**Fig 3:**
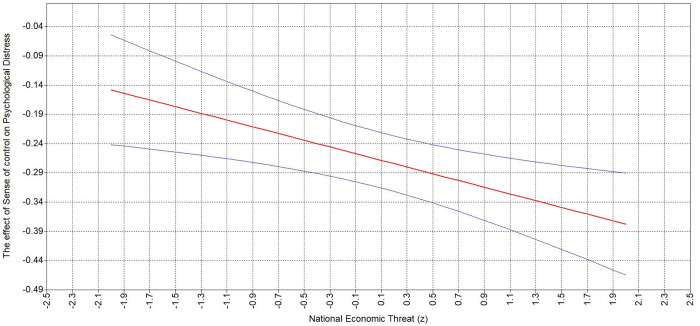
Johnson-Neyman plots depicting interaction between Sense of Control and National Economic Threat.

Hypothesis 3 states that personal (3a) and national (3b) economic threat strengthen the second-stage of the positive indirect relationship between BLM-informed leader behavior and psychological distress. The results indicate a significant interaction of personal economic threat and sense of control (*β *= −.10, **p* *= .001), as well as a significant interaction between sense of control and perceived national economic threat (*β *= −.08, **p* *= .004). Namely, probing of the interaction demonstrates the relationship between sense of control and psychological distress increases with the increase of both personal (Table 5; Fig 2) and national (Table 5a; Fig 3) economic threat.

**Table 5:** Analysis of the Simple Slopes between Sense of Control, Personal and National Economic Threat.

Independent variable	Moderator value	Estimate	95% Boot LLCI	95% Boot ULCI
Personal economic threat	−2SD	−.129	−.215	−.043
	−1SD	−.196	−.254	−.138
	Mean	−.263	−.31	−.215
	+1SD	−.330	−.394	−.266
	+2SD	−.397	−.491	−.303
National economic threat	−2SD	−.148	−.242	−.054
	−1SD	−.205	−.269	−.142
	Mean	−.263	−.310	−.215
	+1SD	−.320	−.379	−.262
	+2SD	−.378	−.465	−.290

Note: *N* = 845.

**Fig 4:**
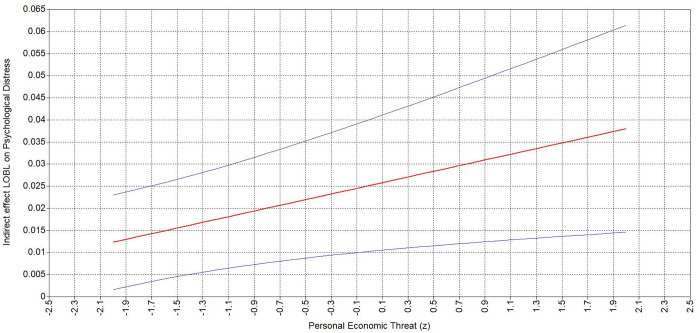
Johnson-Neyman plots depicting indirect effect of BOLB on psychological distress via sense of control moderated by personal economic threat.

**Fig 5:**
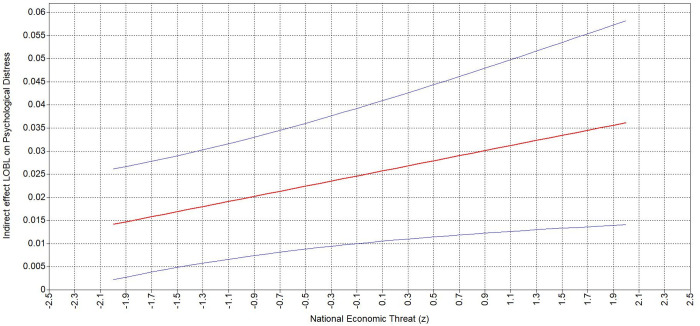
Johnson-Neyman plots depicting indirect effect of BOLB on psychological distress via sense of control moderated by national economic threat.

Examining the conditional indirect effects reveals that when personal (Table 6) and national (Table 6) economic threat are higher (vs. lower), the positive indirect relationship of BLM-informed leader behavior to psychological distress via sense of control is stronger. Specifically, the positive indirect effect of BLM-informed leader on psychological distress was stronger (ΔB = .026, 95% CI = [.006,.047]) at higher level of personal economic threat (B = .038, 95% CI = [.015,.061]; Fig 4) compared to lower level (B = .012, 95% CI = [.002,.023]). Accordingly, the indirect effect was stronger (ΔB = .022, 95% CI = [.003,.042]) at a higher level of national economic threat (B = .036, 95% CI = [.014,.058]) compared to lower national economic threat (B = .014, 95% CI = [.002,.026]; Fig 5). These results support Hypothesis 3a and 3b.

**Table 6:** Analysis of the simple slopes of the indirect effect of BOLB to psychological distress for various levels of perceived and national economic threat.

Independent variable	Moderator value	Estimate	95% Boot LLCI	95% Boot ULCI
Personal economic threat	−2SD	.012	.002	.023
	−1SD	.019	.007	.031
	Mean	.025	.01	.04
	+1SD	.032	.013	.051
	+2SD	.038	.015	.061
National economic threat	−2SD	.014	.002	.026
	−1SD	.020	.007	.032
	Mean	.025	.01	.040
	+1SD	.031	.012	.049
	+2SD	.036	.014	.058

Note: N = 845.

**Fig 6:**
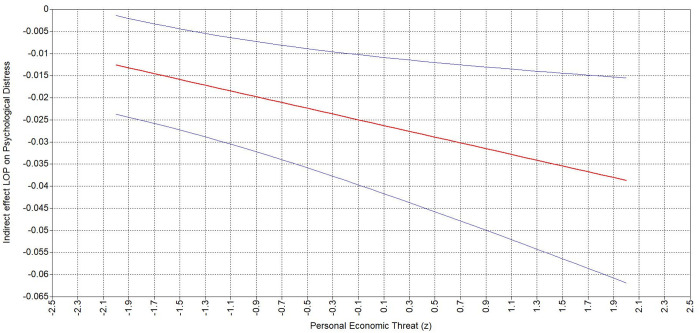
Johnson-Neyman plots depicting indirect effect of POLB on psychological distress via sense of control moderated by personal economic threat.

**Fig 7:**
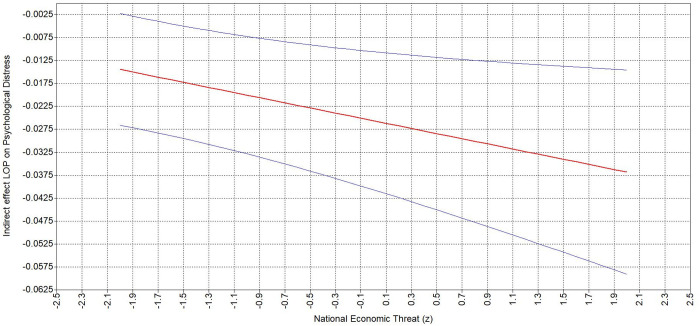
Johnson-Neyman plots depicting indirect effect of POLB on psychological distress via sense of control moderated by national economic threat.

Hypothesis 4 states that personal (4a) and national (4b) economic threat strengthen the second-stage of the negative indirect relationship between people oriented leader behavior and psychological distress. The conditional indirect effect shows that when personal (Table 7) and national (Table 7) economic threat are perceived as high (vs. low), the negative indirect effect of people oriented leader behavior on psychological distress through sense of control is stronger. Specifically, the negative indirect effect of people oriented leader on psychological distress was stronger (ΔB = −.026, 95% CI = [−.045, −.005]) at higher level of personal economic threat (B = −.039, 95% CI = [−.062, −.015]) compared to lower level (B = −.013, 95% CI = [−.024, −.001]; Fig 6). Accordingly, the indirect effect was stronger (ΔB = −.022, 95% CI = [−.041, −.003]) at a higher level of national economic threat (B = −.037, 95% CI = [−.059, −.015]) compared to lower (B = −.014, 95% CI = [−.027, −.002]; Fig 7). These results support Hypothesis 4a and 4b.

**Table 7:** Analysis of the simple slopes of the indirect effect of POLB to psychological distress for various levels of perceived and national economic threat.

Independent variable	Moderator value	Estimate	95% Boot LLCI	95% Boot ULCI
Personal economic threat	−2SD	−.013	−.024	−.001
	−1SD	−.019	−.031	−.007
	Mean	−.026	−.041	−.011
	+1SD	−.032	−.051	−.013
	+2SD	−.039	−.062	−.015
National economic threat	−2SD	−.014	−.027	−.002
	−1SD	−.020	−.033	−.007
	Mean	−.026	−.041	−.011
	+1SD	−.031	−.050	−.013
	+2SD	−.037	−.059	−.015

Note: N = 845.

Because we collected data in several European countries, we offer supplemental analyses performed to further contrast a higher severity cluster comprising Italy and Spain and a lower severity cluster comprising Germany and the Netherlands. These results are presented in S2 Data Attachment. Finally, the data can be found in S3 Data Attachment and the syntax in S4 Data Attachment.

## Discussion

Global crises pose significant challenges and economic threats to individuals and nations alike. In this study, we demonstrated that when a crisis imposes economic strain on employees, the impact of leadership on employee psychological health becomes more pronounced—both in its benefits and costs. Specifically, our findings establish that BLM-informed and people-oriented leadership behaviors have opposing effects on psychological distress, including symptoms of anxiety and depression. These effects are mediated by differences in employees’ sense of control, with people-oriented leadership enhancing control and reducing distress, while BLM-informed leadership diminishing control and exacerbating distress.

### Theoretical implications

By highlighting the opposing psychological health implications of leader behaviors that shows an orientation toward people versus toward the bottom line in a crisis, this study answers calls for research to consider constructive and destructive forms of leadership simultaneously [4,43,106]. In times of economic scarcity, leaders need to act in ways that ensure the survival of their organizations, which might appear to legitimize favoring the bottom line over other priorities, such as employee well-being. Times of crisis can also serve as fruitful ground for destructive forms of leading, as extraordinary circumstances enhance a leader’s ability to exercise authority [107,108]. Simultaneously, the increase in work demands on leaders themselves during a crisis might not leave them with sufficient time and resources to consider the needs of their employees [109,110]. Thus, leaders may think it “makes good business sense” to ramp up the pressure on employees and focus exclusively on the bottom line, especially as doing so might even contribute to favorable performance in the short term [111]. However, in doing so, leaders ignore employee needs and often dismiss ethical practices [34,59]. Our results reveal that, in a crisis, leaders who focus exclusively on the bottom line and behave accordingly hurt employees’ psychological health, especially among employees that are more vulnerable given heightened economic threat. This is problematic because, in the long run, poor employee psychological health is costly for employees, their organizations, and society [7]. In isolating specific leader behaviors, namely offering personalized support (person-oriented) and emphasizing financial targets (BLM-informed), we hoped to maintain conceptual clarity and precision, avoiding conceptual ambiguity and broad operationalization offered by brother leadership styles, e.g., transformational leadership [112,113]

Our findings show that employees’ sense of control at work is a key mechanism through which leaders’ behaviors are linked to employee psychological health. Leaders’ expressions of concern and care for the personal and professional welfare of employees seem to help reduce some of the uncertainty surrounding the crisis and help employees maintain or regain a sense of control. Yet, the leader’s display of the exclusive focus on the bottom line at the expense of other priorities decreases employees’ sense that they can control work events, which in turn relates to their experiencing more worry and other symptoms of anxiety and depression during an already highly uncertain time. Employees’ sense of lacking control at work may also be exacerbated by the toxic working climate that is often fostered by leaders with a bottom line mentality [60]. Previous studies show that, with such leaders, employees can develop a “winner-takes-all” mentality. When this occurs, rather than turning to colleagues as a source of support and collaboration, they start to treat colleagues as “losers” and, desiring to see them fail, may sabotage them, withhold information, or make them look bad in front of the leader [114]. In contrast, providing a safe and healthy environment to employees (i.e., CSR for employees) positively contributes to organizational financial performance [115].

The current study also responds to calls to investigate leadership in context [106,116], in this case during a global crisis. Specifically, we focused on economic threat and showed that experiencing higher economic threat—both personally and at the national level—acts as a moderator of the indirect effect of leadership on employee psychological health. Both personal and national economic threat increase the role of (lacking) a sense of control in employees’ experiencing symptoms of anxiety and depression. Through its influence on employees’ sense of control at work, leadership thus forms a potentially powerful workplace factor that can mitigate or exacerbate psychological health during a global crisis, especially for those under high economic threat who are more vulnerable. Our findings are consistent with work suggesting that leaders have a more prominent role in times of crisis and uncertainty (e.g., [40,55]).

### Practical implications

Our study yields practical insights for both organizations and policy makers. Our findings support the need for comprehensive national governments efforts to mitigate the economic threat accompanying global crises. These efforts are important for the protection of psychological health, especially when crises are long-lasting and induce significant impairments. The COVID-19 pandemic, for instance, caused significant impairments over its long duration, including through containment measures. Our results revealed differences in the average reported symptoms of anxiety and depression of employees between countries, which could be due to the differing levels of strictness in quarantine measures in the course of the pandemic [e.g., [14,117].

Given prior research demonstrating that organizational and societal inequalities reinforce one another, global crises may exacerbate the economic vulnerability of already disadvantaged workers, further compromising their psychological well-being. Employees with fewer individual resources to rely upon [43,118] may be at heightened risk of experiencing negative psychological health outcomes associated with leadership behaviors. Our findings suggest that economically vulnerable employees are particularly susceptible to the psychological consequences of leadership behaviors, underscoring the need for targeted interventions.

To mitigate these risks, employment protection legislation and organizational policies specifically designed to support vulnerable groups may help alleviate economic threat and buffer against adverse health outcomes. While this study focuses on economic threat, future research should explore additional dimensions of vulnerability, such as job precarity, health disparities, and social support systems.

Organizations must equip leaders with the tools to effectively support employees, particularly those in vulnerable economic positions. Evidence-based leadership intervention programs can enhance leaders’ ability to foster employee well-being both during and after crises such as the COVID-19 pandemic. Raising awareness among leaders about the psychological impact of their behaviors may help them reinforce employees’ sense of control at work, provide genuine consideration, and avoid the perception that their focus is solely on financial outcomes, even in times of economic uncertainty.

### Limitations

This study has several limitations that warrant acknowledgment. Data collection occurred during the early stages of the COVID-19 pandemic in the Western world, with initial data gathered in April 2020, followed by two additional waves at two months (Time 2) and eight months (Time 3) later. This longitudinal design provides valuable insights into the role of direct supervisors’ leadership in employee psychological health during crises. However, despite collecting data across three time points and temporally separating the predictor, mediator, and outcome variables, the correlational nature of our study precludes definitive causal inferences. While our hypothesized directionality is grounded in theory, we cannot entirely rule out alternative causal explanations. A fully longitudinal design with multiple time points would be necessary to further clarify the temporal dynamics of these relationships, particularly given the ongoing evolution of the COVID-19 pandemic and its fluctuating impact on health and workplace dynamics.

Additionally, while we separated the measurement time points for our independent, mediator, and dependent variables and demonstrated that our proposed measurement model exhibits superior fit compared to alternatives, future research should incorporate multi-source data and objective financial indicators. Although we tested our moderated mediation model using both subjective and objective indicators of economic threat—yielding consistent findings—incorporating external financial data and multi-source reports would further mitigate the risk of common-method variance [118] and strengthen the robustness of these conclusions.

We collected the data in four European countries, which we clustered based on differences in the severity of threat due to the pandemic despite important similarities. Future research might ascertain the generalizability of the findings in other economic-threat-inducing crises and distinguish better-faring countries from even more negatively affected countries.

## Conclusion

This study highlights the dual impact of leadership during crises, demonstrating that its effects can be both detrimental and beneficial to employee well-being. Specifically, leadership behaviors driven by a bottom-line mentality are positively associated with psychological distress, as they contribute to employees’ diminished sense of control at work. In contrast, people-oriented leadership is negatively associated with psychological distress, as it fosters a greater sense of control among employees. Moreover, our findings indicate that economic threat intensifies the influence of leadership on employee psychological health. The indirect relationship between leadership and psychological distress—mediated by sense of control—becomes more pronounced under higher levels of personal and national economic threat, amplifying the psychological consequences of leadership behaviors during crises.

## Supporting information

S1 Data Attachment – Psychometric properties of leadership measures. (DOCX)

S2 Data Attachment –Comparison across country clusters. (DOCX)

S3 Data Dataset. (CSV)

S4 Data – Syntax. (TXT)
