# Supplementary material for: Prioritizing the bottom line over people in a crisis: How leader behavior affects employee psychological distress under economic threat
Source: PLoS One. 2025 Jul 31;20(7):e0323415. doi: 10.1371/journal.pone.0323415 (PMC12312916; doi:10.1371/journal.pone.0323415)
Supplement: S1 Data — (DOCX) [file pone.0323415.s001.docx]

**Supporting Information**

**S1 Attachment – Psychometric properties of people oriented and bottom-line-mentality informed leader behavior measures**

In an effort to provide further assessment of the psychometric properties of the short forms of the measures used to assess leader behavior, we conducted a supplementary study and evaluated their reliability, factor structure, and the convergent validity between the short and the long forms.

***Procedure and sample***

Data was collected among 55 employees in Germany recruited through online channels. To take part in the study, participants needed to be older than 18 and have a direct supervisor at the time of data collection. Participants were on average 35.98 years old (SD=9.17) and 54.5% declared as women. Moreover, 56.4% participants reported working full-time with a sample average of 33.67 working hours per week (SD=13.63).

***Measures***

People oriented leader behavior was measured using the full 10-item leader consideration subscale from the Leader Behavior Description Questionnaire [LBDQ-XII; 86]. A sample item is: “Looks out for the personal welfare of the team members”. BLM-informed leader behavior was measured using the original 4-item bottom-line mentality scale [19]. A sample item is: “Treats the bottom-line as more important than anything else.” Participants were asked to indicate how strongly they agreed that their direct supervisor demonstrated the two orientations, rating each item on a scale from (1-*strongly disagree* to 7-*strongly agree*).

***Results***

First, for people oriented leader behavior the Cronbach's alpha reliability estimates for the short and long leader consideration scales were .893 and .887, respectively. For BLM-informed leader behavior, Cronbach’s alpha of the shorter bottom-line mentality form was .913 compared to .937 of the longer form. Second, to assess factor structure, we performed a confirmatory factory analysis (CFA) in MPlus. We fitted a two-factor model in which the three items measuring people oriented leader behavior were set to load on the first factor, and the three items measuring BLM-informed leader behavior were set to load on the second factor. The estimated model showed good fit with the data (CFI=1.00, SRMR=.02, RMSEA=.00; χ^2^[8, *N* = 55]= 3.023, *p*= .932) and all items demonstrated high loadings on their respective factors (Table 1A). Third, to assess convergent validity, we computed Pearson correlations between the scores on the short and the long form of both scales in this sample. For people oriented leader behavior (*r* = .933, *p*<. 001) and BLM-informed leader behavior (*r* = .991, *p*<. 001), high correlations indicated high alignment in participants’ responses on the sets of items. In sum, these results provide evidence for the reliability and proposed factor structure of the two shortened leadership measures, as well as their convergent validity with the full-length forms.

***Table S1.*** CFA Standardized factor loadings and scale reliability.

| Construct | Item | Loading | S.E. | Loading/S.E | Reliability |
| --- | --- | --- | --- | --- | --- |
| People oriented leader behavior | Is friendly and approachable. | .86 | .04 | 21.97 | .89 |
|  | Does little things to make it pleasant to be a member of the team. | .80 | .07 | 10.96 |  |
|  | Looks out for the personal welfare of the team members. | 0.95 | .03 | 38.13 |  |
| BLM-informed leader behavior | Only cares about the business. | 0.83 | .05 | 17.28 | .91 |
|  | Treats the bottom-line as more important than anything else. | .84 | .05 | 17.43 |  |
|  | Cares more about profits than employee well-being. | .98 | .03 | 35.15 |  |
