# Supplementary material for: Prioritizing the bottom line over people in a crisis: How leader behavior affects employee psychological distress under economic threat
Source: PLoS One. 2025 Jul 31;20(7):e0323415. doi: 10.1371/journal.pone.0323415 (PMC12312916; doi:10.1371/journal.pone.0323415)
Supplement: S2 Data — (DOCX) [file pone.0323415.s002.docx]

**Supporting Information**

**S2 Attachment –Comparison across country clusters**

Because we collected data in several European countries, we perform supplemental analyses to contrast a higher severity cluster comprising Italy and Spain and a lower severity cluster comprising Germany and the Netherlands, as these two clusters differ in terms of severity of being hit by the spread of the virus as well as in their economic conditions prior to and during the pandemic.

To ascertain that the two country clusters are indeed different, we compared them using independent sample t-tests. The results (Table S1) indicate statistically significant differences in the mean levels of most variables. Consistent with the higher severity of the pandemic, participants in Italy and Spain perceived higher levels of BLM-informed leader behavior, financial and economic threat, psychological distress, and lower people oriented leader behavior and sense of control. Moreover, employees from high-severity cluster indicated that they have been working fewer working hours (M=28.13, SD=13.09) since the onset of the pandemic than participants in Germany and the Netherlands (M=31.51, SD=11.30).

**Table S1.** Descriptive statistics and differences across Low Severity (Netherlands and Germany) and High Severity (Italy and Spain) country clusters.

| Variable | All Participants | | Low-severity Cluster | | High-severity Cluster | | F Estimate (1,843) |
| --- | --- | --- | --- | --- | --- | --- | --- |
|  | *M* | *SD* | *M* | *SD* | *M* | *SD* |  |
| Gender | 0.51 | 0.50 | 0.48 | 0.50 | 0.55 | 0.50 | 3.74 |
| Childcare | 0.37 | 0.48 | 0.28 | 0.45 | 0.46 | 0.50 | 32.50*** |
| Age | 46.76 | 10.67 | 47.57 | 11.78 | 45.91 | 9.33 | 5.20* |
| Education | 2.37 | 0.96 | 2.34 | 1.01 | 2.40 | 0.91 | 0.91 |
| Risk group | 0.22 | 0.41 | 0.22 | 0.42 | 0.21 | 0.41 | 0.08 |
| Contract type | 0.28 | 0.45 | 0.44 | 0.50 | 0.11 | 0.32 | 130.54*** |
| Position | 0.40 | 0.49 | 0.34 | 0.47 | 0.47 | 0.50 | 15.77*** |
| Working from home | 0.43 | 0.50 | 0.49 | 0.50 | 0.37 | 0.48 | 11.19*** |
| Working hours | 29.85 | 12.32 | 31.51 | 11.30 | 28.13 | 13.09 | 16.35*** |
| Leader gender | 0.38 | 0.49 | 0.46 | 0.50 | 0.31 | 0.46 | 20.70*** |
| Frequency of contact | 3.05 | 1.46 | 3.05 | 1.43 | 3.04 | 1.48 | 0.01*** |
| Tenure Job | 11.36 | 9.23 | 10.35 | 9.44 | 12.40 | 8.90 | 10.71** |
| Tenure Leader | 6.48 | 6.51 | 5.80 | 6.40 | 7.19 | 6.55 | 9.91** |
| People oriented leader behavior | 4.93 | 1.48 | 5.26 | 1.35 | 4.57 | 1.53 | 48.80*** |
| BLM-informed leader behavior | 3.14 | 1.50 | 2.79 | 1.43 | 3.50 | 1.49 | 50.87*** |
| Financial threat | 2.49 | 0.90 | 2.31 | 0.84 | 2.68 | 0.92 | 38.48*** |
| Economic threat | 4.92 | 0.96 | 4.73 | 0.96 | 5.11 | 0.93 | 33.51*** |
| Sense of control | 4.29 | 1.42 | 4.43 | 1.39 | 4.15 | 1.45 | 8.15*** |
| Psychological Distress | 1.74 | 0.70 | 1.63 | 0.64 | 1.86 | 0.74 | 23.10*** |

*Note. N*=845*; N_Low severity cluster_=436, N_High severity cluster_=* *419.***p<.001*. Gender was coded as 0=Male; 1=Female. Contract was coded as 0=Permanent; 1=Temporary. Position was coded as 0=Non-managerial; 1=Managerial. Belonging to a Risk group, Childcare, and Working from home were coded as 0=No; 1=Yes.

In addition, we performed a multi-group analysis to test whether the hypothesized relationships varied as a function of the two country clusters (Table S2). First, we tested the fit of the unconstrained multi-group model. Second, we imposed equality constraints on all regression paths. We used the Satorra-Bentler scaled χ2 difference test model test to evaluate changes in model fit. The fully constrained model did not fit the data worse than the unconstrained model (ΔSB-χ2 = 42.99, df = 35, p = .166) indicating no substantial differences across the two clusters (Table S3).

**Table S2.** Results of Multigroup model comparison between the Low Severity (Netherlands and Germany) and High Severity (Italy and Spain) country cluster.

| Models | SB-*χ2* | *df* | SB-SCF | RMSEA | CFI | SRMR | *ΔSB-χ2/df* |
| --- | --- | --- | --- | --- | --- | --- | --- |
| Multigroup Model _Unconstrained_ | 17.230 | 8 | 1.77 | . 05 | .977 | . 03 | *-* |
| Multigroup Model _Fully constrained_ | 61.163 | 43 | 1.18 | .03 | .954 | .04 | 42.99/35 |

Note: *N*=845.
